# Round the Hospitals

**Published:** 1916-03-25

**Authors:** 


### Round the Hospitals.

IN a letter we addressed to the matrons of many hospitals recently we asked for two things. First, whether some member of the nursing staff, probably a sister, possibly a staff-nurse who takes a genuine interest in her work and patients, would kindly act as our correspondent, so that the institution in which they usefully laboured might not bury its light under a bushel. We have already dealt with the sisters who found themselves too busy and occupied to entertain this suggestion with favour. This week another difficulty has been advanced owing to the shortage of sisters, many of whom are serving with the Forces, and the desire to keep their posts open until after the war. In hospitals where these difficulties are genuinely present an opening is afforded to the staff nurses and the sisters *pro tem.*, or, failing these, might not the interesting duty of correspondent to THE HOSPITAL be undertaken by some V.A.D., being an educated woman, who probably may have acquired the knack of descriptive writing, though she may not be fully conscious of it until she elects to co-operate with the Editor by filling this position. Each correspondent could so bring aid to and arouse public interest in the hospital which has given her an opening to do war service. In this connection it is well to realise that the Editor cannot sit in his office and evolve hospital paragraphs which will meet the difficulty, but where neither the matron nor the sisters nor the staff-nurse can for some reason become a correspondent, there the "V.A.D.," when selected by the matron, might make her acknowledgments to the hospital authorities who have admitted her to training by filling the vacancy during the war.

The second request which we made was that each matron should kindly send us a photograph of herself, and, if she has one, of her staff, that we may have at The Hospital office an album which will contain the portraits of all the women who are acting as matrons of a voluntary, a military, a territorial, or a Red Cross hospital at the time of the Great War. We have already received with gratitude some portraits for this purpose, and we mention the subject again because at the conclusion of the war, and for many years afterwards, such an album would have an abiding interest, and in due course may one day find its way to the British Museum as a national relic of historical interest and value. Short biographies might be sent with each photograph. We are bold enough to hope that few or no matrons will find it possible to resist the Editor's appeal in this matter, or fail to do their part to carry out the idea and make it entirely worthy of the profession of nursing in the United Kingdom. Then, as we hope, the idea may also commend itself to many matrons throughout the Empire.

We take this opportunity of thanking the large number of matrons who have been good enough to write to us to say complimentary things about this department of the paper. It is a matter of pride that in the thirtieth year of its existence we find through our correspondence that the matron of practically every important and actively managed voluntary hospital is a constant reader of The Hospital. This knowledge, resting as it does upon documentary evidence spontaneously supplied by the matrons themselves, makes us increasingly wishful that The Hospital, so far as nursing matters are concerned, should be kept well up to date and meet to the utmost the wishes and wants of individual matrons and sisters everywhere. We hope, indeed, that every matron may continue to regard The Hospital as her friend and helper. May time prove that not a single one of them ever fails to write to the Editor when information on some point is desired, or when there may be some ambition ungratified, or the desire to extend individual knowledge by foreign travel, or the efficiency of the nursing department or training school for which they are responsible.

The war has had the effect of transferring to matrons and sisters many duties usually performed by the medical staff of hospitals. Exception is taken in last week's issue of the *Lancet* to the practice of habitually allowing a matron to give anæsthetics. Our contemporary holds the view that only a qualified medical man or woman is capable of taking the great responsibility of administering anæsthetics. Every matron will do well to remember that if a fatality were to occur when she was administering the anæsthetic the responsibility would rest with the surgeon who had entrusted her with the work.

It is, however, a fact that in France and America trained nurses are recognised as qualified for this work, and do it with signal success. In the Mayo Hospital at Rochester, U.S.A., where the Mayo Brothers perform world-famed operations, all anæsthetics have for many years past been administered by the nuns, who are trained nurses; and it is recorded that amongst the thousands of operations performed at this hospital no death under an anæsthetic has ever occurred. In view of the great shortage of medical men at the present time, opportunity to become proficient in the administration of anæsthetics has been afforded to experienced nurses in some hospitals already, which has enabled them to undertake this work to the satisfaction of the practitioner and with success.

The following story of a soldier's impressions of war nursing shows how unexpectedly different is their point of view from ours. A girl was recounting the experiences of her young brother, who had returned wounded from the Dardanelles. She told of how, never before having seen death or illness, he had to advance over the fallen bodies of his comrades, and of the unutterable horror of it. When, having been wounded, he was conveyed to a dressing station, she related how two nurses who had been sent ashore for the purpose were busy injecting morphia to those men who were in great pain; "they just went on with their work and never smiled. If only they had smiled it might have cheered the poor chaps up," said the girl. "My brother said it made everything seem more dreadful seeing the nurses with such set, stern faces. The thought that the scene which shocked him so much might have made smiling difficult to the nurses, still less that a cheery remark might have seemed out of place at such a time, when death was hovering so near, does not seem to have occurred to the boy, and he probably expressed what not only his comrades, but the great majority of our soldiers feel. They show it in the astonishing devotion to the gramophone which is so evident in our military hospitals. Perhaps those nurses, who are keenest to help and show most sympathy for all our men are suffering, are those who most need reminding that both officers and men are alike in their dislike of pity, and that they love a touch of humour even when things are at their worst.

*** It is our invariable practice to pay for all items of news which we may publish. The minimum payment is 5s. Paragraphs of about twenty lines in length—that is to say, of 150 words or less—are preferred. In this section during the war we propose to give 10s. to the correspondent who may send us in any week the best incident connected with hospital life and work. In this way we hope to afford to all practical aid and encouragement; while those who reciprocate will be able to render us invaluable service. Contributions should be addressed to The Editor, The Hospital, 28 and 29 Southampton Street, Strand, London, W.C., and, to be in time for the current week's issue, should reach him by the first post on Mondays.

